# Knowledge, Attitude, and Practices of Food Handlers on Food Safety and Personal Hygiene During Arbaeenia Mass Gathering, Baghdad, Iraq, 2014: Cross-Sectional Study

**DOI:** 10.2196/10922

**Published:** 2019-10-09

**Authors:** Faris Lami, Firas Radhi, Safauldeen Al Dahhan, Rana Adel Hashim, Hussein Mahmood, Rawan Araj, Ali Arbaji

**Affiliations:** 1 Department of Community and Family Medicine College of Medicine University of Baghdad Baghdad Iraq; 2 Iraq Ministry of Health Baghdad Iraq; 3 Global Health Development Amman Jordan

**Keywords:** food, hygiene, knowledge, attitudes, Iraq

## Abstract

**Background:**

Millions of pilgrims attend Arbaeenia mass gathering (MG) in Iraq each year. Thousands of individuals work voluntarily at temporary rest areas (locally called Mawakib), distributed in most of Iraq governorates, to provide food and other services to the MG attendees. The potential for improper handling of food at Mawakib increases the risk of waterborne and foodborne diseases.

**Objective:**

This study was aimed to assess the knowledge, attitude, and practices (KAP) of food handlers in Mawakibs in Baghdad city during Arbaeenia MG.

**Methods:**

A random sample of 100 Mawakibs was selected in Baghdad, 50 from the eastern side (Rusafa) and 50 from the western side (Kerkh), and five food handlers were randomly selected from each Mawakib. A questionnaire was used to collect demographic data and KAP for food safety and personal hygiene. The questionnaire included 25 questions addressing knowledge, 10 addressing attitudes, and 14 addressing practices of the food handlers with respect to food safety and personal hygiene. Questions on knowledge and attitudes were answered through direct interview with the food handlers, whereas the questions on practices were answered through direct observation while handling or serving the food. SPSS version 20 (IBM SPSS Statistics 20) was used for data analysis and describing proportions.

**Results:**

There was a varied knowledge of food safety practices among the individuals interviewed. On a scale of 3, the overall average score for both the attitude and practices for food safety and personal hygiene was 2, which corresponds to fair attitude and practices. The attitudes varied significantly by location, age group, and education, whereas the practices varied by location, age groups, employment, and previous experiences.

**Conclusions:**

The food handlers had unsatisfactory attitudes and practices toward food handling and personal hygiene. Their participation in food handling at Mawakib carries a potential risk of spreading foodborne and waterborne diseases. All individuals intending to serve in Mawakib as food handlers should be licensed from the Ministry of Health after completing a formal training in food safety and personal hygiene.

## Introduction

### Background

Mass gatherings (MGs) are defined as the congregation of as few as 1000 individuals to upwards of 25,000 in a specific location for a designated period [1]. Such occurrences put a strain on the local resources, such as food, drinks, accommodation, and health care, and can pose a health risk to the population. Infectious diseases, such as foodborne and waterborne diseases, are considered a major public health concern during MGs, along with road traffic accidents, other injuries, and terrorism [2]. However, limited data are available on foodborne and waterborne disease outbreaks during MGs, largely because of poor health surveillance systems and limited research on population health during MGs.

Poor food safety and personal hygiene are the main risk factors for foodborne and waterborne illnesses; food handlers play an important role with regard to these risk factors [3]. Contaminated hands are vehicles for enteric virus transmissions, and food preparation and handling affect the safety of the food served [4]. In Iraq, religious MGs occur throughout the year, mainly in Karbala, Najaf, and Baghdad. Arbaeenia is the largest religious MG that annually convenes in Karbala, where more than 10 million people, mainly from Iraq, visit Imam Hussein’s shrine in Karbala. Attendees walk hundreds of kilometers for many days to reach Karbala city.

Faith-based organizations and the general public are the main providers of water, food, shelter, accommodation, and, to a certain extent, health services in rest areas for pilgrims, locally called Mawakibs. Most of the individuals preparing and serving food are not certified food handlers, and Mawakibs are not regularly inspected by health authorities. The number of Mawakibs providing different services during the MGs has greatly increased over the last few years [5].

### Objectives

The objective of this study was to assess the knowledge, attitude, and practices (KAP) of Mawakib food handlers with respect to food safety and personal hygiene during the Arbaeenia MG in Baghdad, Iraq, 2014.

## Methods

### Study Design

To assess the KAP of Mawakib food handlers with respect to food safety and hygiene, we conducted this cross-sectional study in Baghdad during the Arbaeenia MG, December 5 to December 9, 2014. A simple random sample of 100 Mawakibs was selected from a list, which included the names and locations of these Mawakibs; 50 from Rusafa (eastern side of Baghdad) and 50 from Kerkh (western side of Baghdad). A total of 5 food handlers were selected from each Mawakib to participate in the study. Overall, 8 surveyors were trained to collect the data. The study was approved by the research committee of Iraq’s Ministry of Health. The Mawakibs were selected after obtaining a list of all the Mawakibs and assigning them a sequential number to draw a random sample according to the calculated sample size.

### Questionnaire

A questionnaire was used to collect data on the 3 domains of KAP. The questionnaire also collected data on sociodemographics and previous experience in food handling. Previous experience was defined as any former serving or food handling during similar events and MGs. The questionnaire was translated from English into Arabic (local dialect) and translated back to English to ensure accurate translation.

The questionnaire had 49 questions in total. Overall, 25 questions addressed the knowledge of food safety and personal hygiene. Furthermore, 10 questions addressed the attitudes toward food safety and personal hygiene habits while handling and serving food. A total of 14 questions addressed the practices of food handlers on food safety and hygiene when serving food to the visitors of the Mawakib during Arbaeenia MG. The knowledge and attitude sections of the questionnaire were completed through face-to-face interview, during which the data collectors verbally asked the questions to the study participants and then recorded their answers. The practices section of the questionnaire was completed by the data collectors based on their observations of the food handling practices.

The knowledge section of the questionnaire required *yes* or *no* responses. The practices and attitude sections required a response of *always*, *sometimes*, *rarely*, or *never* /*refused*. We assigned a score of 3 to *always*, a score of 2 to *sometimes*, a score of 1 to *rarely*, and 0 to *never*. However, *refused* and missing responses were excluded from the analysis.

### Data Analysis

Data were coded and entered into Epi Info 7 (CDC's Epi Info 7) and SPSS version 20 (IBM Corp, Armonk, NY, USA) was used for data analysis. The percentage of food handlers who correctly answered the questions was calculated in the knowledge domain. For the questions on attitude and practices, the average scores were calculated for the responses to each question, in addition to the overall average score. The attitude and practices sections were categorized into poor, fair, and good, corresponding to the average scores of <1.5, 1.5 to 2.4, and 2.5 to 3, respectively.

The percentage of respondents with poor, fair, or good attitudes and practices was estimated by sociodemographics. Chi-square tests and Fisher exact tests were used to test the association between KAP and sociodemographics. A *P* value of less than .05 was considered statistically significant.

## Results

### Food Handlers’ Characteristics

A total of 504 food handlers were interviewed. One data collector interviewed 6 instead of 5 food handlers in 4 Mawakibs. The food handlers were predominately male, in total 498 (98.8%, 498/504). The average age of the food handlers was 37 (SD 12) years. Furthermore, 50.4% (254/504) had secondary or postsecondary education. Only 3.2% (16/504) were basically full-time food handlers (Table 1).

**Table 1:** Distribution of the food handlers during Arbaeenia mass gathering in Baghdad, Iraq, by sociodemographic characteristics and previous experience in food handling (N=504).

Sociodemographic characteristics		Statistics, n (%)
**Sex**		
	Female	6 (1.2)
	Male	498 (98.8)
**Location**		
	Karkh	252 (50.0)
	Rusafa	252 (50.0)
**Education level**		
	Illiterate	58 (11.5)
	Presecondary	192 (38.1)
	Secondary	161 (31.9)
	Postsecondary	93 (18.5)
**Employment**		
	Full-time food handler	16 (3.2)
	Unemployed or retired	14 (2.8)
	Full-time employee	131 (25.9)
	Student	62 (12.3)
	Day laborer	281 (55.8)
**Previous experience in food handling**		
	No	143 (28.4)
	Yes	361 (71.6)

### Knowledge

The percentage of food handlers who correctly answered questions related to food safety and personal hygiene ranged from 8% to 100%. Poor knowledge was mainly related to storing food using ice baths, contaminating food with watches and rings that were worn, and food poisoning from eating perishables left out (Table 2).

Knowledge scores of the study participants were categorized into 3 categories: *poor*, *fair*, and *good*. The results of this score categorization and the statistical association with background variables are shown in Table 3. Knowledge of food handlers about food safety and personal hygiene was significantly associated with location (*P*=.001) and job (*P*=.04); full time employees had the best knowledge scores.

**Table 2.** Responses to questions on knowledge by the study participants (N=504).

Number	Knowledge items	Correct answer, n (%)
1	It is OK to mix raw meat with processed ones.	41 (8.1)
2	Consumption of food in any container can be delayed.	44 (8.7)
3	Ice baths—used as cooling systems—are not safe to store food for a prolonged time.	167 (33.5)
4	Wearing rings and watch can contaminate food.	396 (78.6)
5	Eating perishables left out for more than 2 hours at room temperature can lead to food poisoning.	396 (78.7)
6	Multiple food tastings from the same dish can contaminate the dish with contagious microbes.	403 (80.3)
7	Multiple people drinking from the same cup can contaminate the cup with contagious microbes.	407 (82.9)
8	Certain ways of handling food can cause food poisoning and encourage the growth of bacteria, viruses, and parasites	426 (84.7)
9	It is essential for food handlers to have a dry storage unit.	422 (85.3)
10	Food handler with a cold can contribute to the spread of diseases while handling food.	431 (85.7)
11	Infected wound can increase the risk of food poisoning while handling food.	448 (88.9)
12	Food handler with a fever can contribute to the spread of diseases while handling food.	452 (89.7)
13	Open sores can contribute to the spread of diseases while handling food.	462 (91.7)
14	Vaccination is important to prevent the spread of diseases.	466 (93.3)
15	Washing body frequently is important.	469 (94.6)
16	Smoking is a bad practice while handling food at Mawakibs.	481 (95.6)
17	Food handlers should avoid preparing food if they are suffering from vomiting.	487 (96.6)
18	Proper food covering can help prevent contamination.	488 (96.8)
19	Food handlers should avoid preparing food if they are suffering from diarrhea.	493 (97.8)
20	Food preparation must be done using clean equipment.	493 (97.8)
21	Tidying hair and trimming finger nails contribute to your health positively.	494 (98.0)
22	It is a good practice to use disposal utensils when serving food.	495 (98.2)
23	The cooking equipment should be washed immediately after use.	496 (98.6)
24	Washing hands should be a frequent practice.	501 (99.4)
25	Food preparation must be done using potable water.	503 (99.8)

**Table 3:** Distribution of study participants by knowledge levels and background variables.

Background variables		Knowledge levels			
		Poor, n (%)	Fair, n (%)	Good, n (%)	*P* value
**Location**					**.001**
	Karkh (N=252)	2 (0.8)	30 (11.9)	220 (87.3)	
	Rusafa (N=252)	24 (9.5)	117 (46.4)	111(44.0)	
**Age group (years)**					**.22**
	<20 (N=30)	3 (10)	13 (43)	14 (47)	
	20-39 (N=275)	15 (5.5)	71 (25.8)	189 (68.7)	
	40-59 (N=181)	7 (3.9)	56 (30.9)	118 (65.2)	
	≥60 (N=18)	1 (6)	7 (39)	10 (56)	
**Education level**					**.57**
	Illiterate (N=58)	2 (3)	21 (36)	35 (60)	
	Subsecondary (N=192)	13 (6.8)	55 (28.6)	124 (64.6)	
	Secondary (N=161)	9 (5.6)	43 (26.7)	109 (67.7)	
	Higher education (N=93)	11 (12)	16 (17)	76 (82)	
**Job**					**.04**
	Full-time food handler (N=16)	1 (6)	8 (50)	7 (44)	
	Unemployed/retired (N=14)	0 (0)	4 (29)	10 (71)	
	Full-time employee (N=131)	3 (2.3)	18 (13.7)	110 (83.9)	
	Student (N=62)	4 (6)	16 (26)	42 (68)	
	Day laborer (N=281)	8 (2.8)	52 (18.5)	221 (78.6)	
**Previous experience**					**.09**
	No (N=143)	7 (4.9)	32 (22.4)	104 (72.7)	
	Yes (N=361)	19 (5.3)	115 (31.9)	227 (62.9)	

### Attitude

On a 3-point scale, the overall average score of the questions on attitude was 2. A total of 6 questions had an average score of 2, and 4 questions had 3. The distribution of responses to attitude questions is shown in Table 4.

The overall attitude of food handlers toward food safety and personal hygiene while handling and serving food was scored as fair.

Attitude scores of the study participants were categorized into 3 categories: *poor*, *fair*, and *good*. The results of this score categorization and the statistical association with background variables are shown in Table 5. Attitude scores were significantly associated with location (better in Kerkh; *P*=.001), age (*P*=.01), education (*P*=.008), and previous experience with food handling (*P*=.03).

**Table 4:** Distribution of responses to questions on attitude.

Number	Attitude items	Always, n (%)	Sometimes, n (%)	Rarely, n (%)
1	Staying home instead of going to Mawakib to serve food when having vomiting	373 (74.0)	81 (16.1)	50 (9.9)
2	Staying home instead of going to Mawakib to serve food when having diarrhea	373 (74.0)	81 (16.1)	50 (9.9)
3	Staying home instead of going to Mawakib to serve food when having open sores	383 (76.0)	66 (13.1)	55 (10.9)
4	Not wearing rings and watch during food handling	373 (74.0)	96 (19.1)	35 (6.9)
5	Staying home instead of going to Mawakib to serve food when having an infected wound	393 (78.0)	61 (12.1)	50 (9.9)
6	Staying home instead of going to Mawakib to serve food when having a cold	403 (80.0)	61 (12.1)	40 (7.9)
7	Encourage food handlers to get vaccinated	428 (84.9)	45 (8.9)	31 (6.2)
8	Tidying hair and trimming finger nails	423 (83.9)	76 (15.1)	5 (1.0)
9	Washing body	443 (87.9)	55 (10.9)	6 (1.2)
10	Washing hands as necessary	454 (90.1)	45 (8.9)	5 (1.0)

**Table 5:** Distribution of the study participants by attitude levels and background variables.

Background variable		Attitude levels			*P* value
		Poor, n (%)	Fair, n (%)	Good, n (%)	
**Location**					**.001**
	Kerkh (N=252)	66 (26.2)	100 (39.7)	86 (34.1)	
	Rusafa (N=252)	112 (44.4)	103 (40.9)	37 (14.7)	
**Age group (years)**					**.01**
	<20 (N=30)	13 (43)	10 (33)	7 (23)	
	20-39 (N=275)	77 (28.0)	122 (44.4)	76 (27.6)	
	40-59 (N=181)	82 (45.3)	64 (35.4)	35 (19.3)	
	≥60 (N=18)	6 (33)	7 (39)	5 (28)	
**Education level**					**.008**
	Illiterate (N=58)	15 (26)	19 (33)	24 (41)	
	Subsecondary (N=192)	77 (40.1)	83 (43.2)	32 (16.7)	
	Secondary (N=161)	58 (36.0)	60 (37.3)	43 (26.7)	
	Higher education (N=93)	28 (30)	41 (44)	24 (26)	
**Job**					**.11**
	Full-time food handler (N=16)	2 (13)	11 (69)	3 (19)	
	Unemployed or retired (N=14)	8 (57)	6 (43)	0 (0)	
	Full-time employed (N=131)	45 (34.4)	53 (40.5)	33 (25.2)	
	Student (N=62)	24 (39)	26 (42)	12 (19)	
	Day laborer (N=281)	99 (35.2)	107 (38.1)	75 (26.7)	
**Previous experience**					**.03**
	No (N=143)	59 (41.3)	60 (41.9)	24 (16.8)	
	Yes (N=361)	119 (32.9)	143 (39.6)	99 (27.4)	

### Practices

The overall average score for responses on practices for food safety and personal hygiene was 2; the average score of 7 of the questions was 2 and the remaining 7 averaged a score of 3 (Table 6). The overall practice of food handlers was therefore fair.

Practice scores of the study participants were categorized into 3 categories: *poor*, *fair*, and *good*. The results of this score categorization and the statistical association with background variables are shown in Table 7. The practice scores were significantly associated with the location (better in Kerkh; *P*=.001) and previous experience with food handling (*P*=.001).

**Table 6:** The distribution of responses to questions on practices.

Number	Practice items	Always, n (%)	Sometimes, n (%)	Rarely, n (%)	No response, n (%)
1	Not serving food for visitors, which has been kept in containers for a long time	359 (71.2)	55 (10.9)	30 (6.0)	60 (11.9)
2	Not using ice baths as a cooling system to store food for a prolonged time	277 (55.0)	76 (15.0)	10 (2.0)	141 (28.0)
3	Using dry storage unit to store food	418 (83.0)	40 (7.9)	15 (3.0)	81 (16.1)
4	Not allowing multiple visitors to taste food from the same dish	328 (65.0)	35 (6.9)	10 (2.0)	131 (26.0)
5	Not allowing multiple visitors to drink from the same cup	328(65.0)	30 (6.0)	10 (2.0)	136 (27.0)
6	Not smoking while handling food at Mawakib	333 (66.0)	35 (7)	5 (1)	131 (26)
7	Not reheating the food more than once before serving it	337 (67.0)	30 (6.9)	45 (8.9)	91 (18.1)
8	Using disposal units to serve food for visitors	343 (68.0)	25 (5.0)	5 (1.0)	131 (26.0)
9	Not serving perishables left out for more than 2 hours at room temperature for visitors	338 (67.0)	35 (6.9)	5 (1.0)	126 (25.0)
10	Washing cooking equipment immediately after use	358 (71.0)	10 (2.0)	5 (1.0)	131 (26.0)
11	Covering food properly to prevent contamination	358 (71.0)	10 (2.0)	5 (1.0)	131 (26.0)
12	Preparing food using potable water	358 (71)	15 (3.0)	2 (0.4)	129 (25.6)
13	Separating raw meat from processed ones	363 (72.0)	10 (2.0)	5 (1.0)	126 (25.0)
14	Preparing food using clean equipment	368 (73.0)	9 (1.8)	1 (0.2)	126 (25.0)

**Table 7:** Distribution of the study participants by practice levels and background variables.

Background variables		Practice levels			
		Poor, n (%)	Fair, n (%)	Good, n (%)	*P* value
**Location**					**.001**
	Kerkh (N=252)	58 (23.0)	70 (27.8)	124 (49.2)	
	Rusafa (N=252)	119 (47.2)	90 (35.7)	43 (17.1)	
**Age group (years)**					**.08**
	<20 (N=30)	13 (43)	8 (27)	9 (30)	
	20-39 (N=275)	83 (30.2)	88 (32.0)	104 (37.8)	
	40-59 (N=181)	75 (41.4)	60 (33.1)	46 (25.4)	
	≥60 (N=18)	6 (33)	4 (22)	8 (44)	
**Education level**					**.05**
	Illiterate (N=58)	16 (28)	20 (34)	22 (38)	
	Subsecondary (N=192)	54 (28.1)	70 (36.5)	68 (35.4)	
	Secondary (N=161)	65 (40.4)	47 (29.2)	49 (30.4)	
	Higher education (N=93)	42 (45)	23 (25)	28 (30)	
**Job**					**.06**
	Full-time food handler (N=16)	3 (19)	8 (50)	5 (31)	
	Unemployed or retired (N=14)	6 (43)	6 (43)	2 (14)	
	Full-time employed (N=131)	38 (29.0)	40 (30.5)	53 (40.5)	
	Student (N=62)	31 (50)	15 (24)	16 (26)	
	Day laborer (N=281)	99 (35.2)	91 (32.4)	91 (32.4)	
**Previous experience**					**.001**
	No (N=143)	33 (23.1)	43 (30.1)	67 (46.9)	
	Yes (N=361)	134 (39.9)	117 (32.4)	100 (27.7)	

## Discussion

### Principal Findings

The study shows that the food handlers serving Arbaeenia MG attendees had satisfactory knowledge, fair attitudes, and fair practices with respect to food safety and personal hygiene. According to sociodemographics, the attitudes and practices of the food handlers varied.

Correct knowledge regarding food safety and personal hygiene varied among the food handlers. A lack of knowledge on food safety measures, such as proper preparation and storage, carries the risk for potential events of foodborne diseases. Similar findings of lack of knowledge were present in previously conducted KAP studies of food handlers [6,7]. Mawakib food handlers are volunteers and not all the handlers reported prior experience in food handling, which may explain a lack of knowledge in food safety and personal hygiene. Variation in knowledge may be because of the lack of training and information, as reported in similar studies [8,9].

The findings on the attitudes of the food handlers toward food safety and personal hygiene habits were similar to the findings on the practices. Attitudes have an impact on practices, as observed in other KAP studies [10]. Attitudes and practices varied significantly according to location and age, which suggests that attitudes may have shaped the observed practices of the food handlers. The results of this study indicated that the lowest education level (illiterate) had a higher percentage of food handlers with good attitudes toward food safety and personal hygiene than all other education levels. In the domain of practices, food handlers with secondary education had the highest percentage of good practices. These findings suggest that there is no strong correlation between the education level and the attitudes and practices of food handlers; similar findings in previous studies suggest that education level does not influence food safety and personal hygiene [11].

### Conclusions

We can conclude that in spite of satisfactory knowledge, the current attitudes and practices of food handlers are not satisfactory; a finding that makes their participation in provision of food for MG attendees in Mawakib a noticeable risk. Iraq’s Ministry of Health is requested to make rigorous standards and licensure mandatory for all individuals intending to serve in Mawakib as food handlers. They have to complete a formal training in food safety and personal hygiene.

The results of this study cannot be generalized on all Mawakibs throughout the country as the study was conducted in only 1 city. Hence, one of the recommendations to the Ministry of Health is to conduct a large-scale survey in all cities affected with the MGs and serving of food to have clear guidance on food safety and the needed regulations.

## Abbreviations

KAP: knowledge, attitude, and practices
MG: mass gathering
